# cMolGPT: A Conditional Generative Pre-Trained Transformer for Target-Specific De Novo Molecular Generation

**DOI:** 10.3390/molecules28114430

**Published:** 2023-05-30

**Authors:** Ye Wang, Honggang Zhao, Simone Sciabola, Wenlu Wang

**Affiliations:** 1 Biotherapeutic and Medicinal Sciences, Biogen, 225 Binney Street, Cambridge, MA 02142, USA; simone.sciabola@biogen.com; 2 College of Agriculture and Life Sciences, Cornell University, Ithaca, NY 14850, USA; hz269@cornell.edu; 3 Computer Science, Texas A&M University-Corpus Christi, 6300 Ocean Dr, Corpus Christi, TX 78412, USA

**Keywords:** molecular design, machine learning, generative pre-trained transformer, GPT

## Abstract

Deep generative models applied to the generation of novel compounds in small-molecule drug design have attracted a lot of attention in recent years. To design compounds that interact with specific target proteins, we propose a Generative Pre-Trained Transformer (GPT)-inspired model for de novo target-specific molecular design. By implementing different keys and values for the multi-head attention conditional on a specified target, the proposed method can generate drug-like compounds both with and without a specific target. The results show that our approach (cMolGPT) is capable of generating SMILES strings that correspond to both drug-like and active compounds. Moreover, the compounds generated from the conditional model closely match the chemical space of real target-specific molecules and cover a significant portion of novel compounds. Thus, the proposed Conditional Generative Pre-Trained Transformer (cMolGPT) is a valuable tool for de novo molecule design and has the potential to accelerate the molecular optimization cycle time.

## 1. Introduction

Small-molecule drug design aims to identify novel compounds with the desired chemical properties. From a computational perspective, we consider this task an optimization problem, where we search for the compounds that will maximize our quantitative goals within the chemical space. However, this optimization task is computationally intractable because of the unbounded search space. Although it has been estimated that the potential number of drug-like molecules ranges from $10^{60}$ to $10^{100}$ [1], only about $10^{8}$ molecules have ever been synthesized [2]. Numerous computational methods, such as virtual screening, combinatorial libraries, and evolutionary algorithms, have been developed to search the vast chemical space *in silico* and *in vitro*. Computational chemistry has reduced the experimental efforts of molecular design and addressed the experimental limitations [3,4,5,6]. Recent works have demonstrated that machine learning, especially deep learning methods, can produce new small molecules [7,8,9,10] with the desired biological activity. In this work, we aim to apply deep learning techniques and incorporate as much chemical domain knowledge as possible to facilitate directed navigation toward the desired locations within the various chemical search spaces.

SMILES strings, which are text strings that each correspond to a specific chemical structure, are popular inputs/outputs in the field of deep learning-based drug discovery. In recent years, many efforts have been made to enable the conditional generation of drug-like molecules (represented as SMILES strings) with specific properties. For example, recent works have shown that a variational autoencoder can generate molecules [11,12] with specific properties by utilizing concatenated SMILES strings containing the property of interest [13]. Additionally, Recurrent Neural Network (RNN)-based generative models have been extensively tested in molecular design [14,15,16,17,18] since an increased number of chemical structures can be sampled using an RNN to learn from a limited set of SMILES strings [19]. One can also potentially sample target-specific chemical structures by fine-tuning an RNN with a small set of active SMILES strings against a specific biological target [17]. Alternatively, RNNs can be further modified by setting the interval states of RNN cells (e.g., LSTM) to produce SMILES strings with specific target properties [20]. Another work instead modifies an existing SMILES string by prepending chemically-informed changes [21]. They then test on both Transformer [22] and RNN seq2seq structures by inputting this modified SMILES string and outputting a SMILES string with the desired properties. In addition to variational autoencoders [13] and conditional recurrent neural networks [20], conditional graph generative models [23] generate molecular graphs instead of SMILES strings with specified conditions, and the conditional representation is added as an additional term within the hidden state of each layer. Reinforcement learning can also further [24,25] confine the chemical space for specific properties.

In this work, we instead rely on Natural Language Processing (NLP) and treat the small-molecule drug design problem as a text/SMILES generation problem. A number of deep learning techniques have been successfully applied to text generation. For example, the GPT (Generative Pre-Trained Transformer) series [26,27,28] uses an autoregressive language model to produce human-like text by training on vast amounts of unlabeled human-written text. The text generated from the GPT model is of high quality and hard to distinguish from human-written content. Similarly, GPT models are able to learn chemical structures from a large molecular dataset. The general idea of GPT [26,27] is to learn natural language by predicting the next word given the previous words from a large text corpus using unsupervised learning. The unsupervised pre-training is able to prime the model with drug-like knowledge and enforce valid SMILES strings. The well-trained GPT is able to generate synthetic text excerpts while conditioning on an arbitrary input. Similarly, the GPT structure is able to support conditional generations by fine-tuning small-sized supervised data.

In the scope of small-molecule drug design, it is essential to enable the generations to be guided by predefined conditions such as the target protein. In this study, we formulate the molecular design problem as a conditional sequential generation given the target protein and propose a conditional Transformer architecture (cMolGPT) that auto-regressively generates target-specific compounds. We first propose to pre-train a Transformer-based auto-regressive decoder on the MOSES [11] dataset, without target information (denoted as the base model), and introduce randomness into the sampling process to generate more variations and make the trained model more “creative”. As we can prompt the Transformer paradigm with different embeddings as keys and values to enforce the generative process to be conditioned on the specified targets, the base model is fine-tuned on three target-specific datasets (EGFR, HTR1A, and S1PR1). Our generative pre-training guarantees that the generated sequence will be a valid drug-like structure in compliance with the SMILES grammar, and the Transformer structure also supports conditional generations by directed navigation toward the specified target in the chemical space. The results show that the proposed Transformer is capable of generating novel chemical matter specifically designed to hit a target of interest, with the inherent limitation that the generated compounds will be somewhat similar to the compounds in the training set. Our proposed Conditional Generative Pre-Trained Transformer (cMolGPT) is a valuable tool for de novo molecule design and has the potential to dramatically accelerate the molecular optimization cycle time.

## 2. Results and Discussion

We quantitatively assessed the performance of our generative model through two tasks: (a) generating compound libraries using a pre-trained base model of cMolGPT to evaluate its capability of creating novel and diverse virtual compound libraries, and (b) generating target-specific molecules, which are often representative of early-stage drug discovery, using cMolGPT.

### 2.1. Generating Compound Libraries Using Pre-Trained cMolGPT

We evaluated the generated compounds based on the various aspects of molecule generation proposed in [11], including the fraction of valid (Valid) and unique molecules, Unique@1K, Unique@10K, fragment similarity (Frag), and similarity to the nearest neighbor (SNN). Besides basic metrics, we compared the distribution of drug-likeness properties (LogP, molecular weight, QED, and synthetic accessibility score ) between generated and real compounds. The performance was reported on 30,000 molecules generated from each generative model. We computed all metrics (except for validity) only for valid molecules from the generated set. We compared our method with the eight different baseline models, including the Hidden Markov Model (HMM) [29], N-gram generative model [11], combinatorial generator (Combinatorial) [11], character-level recurrent neural network (CharRNN) [17], SMILES variational autoencoder (VAE) [30], adversarial autoencoder (AAE) [31], junction tree VAE (JTN-VAE) [32], and latent vector-based generative adversarial network (LatentGAN) [33].

**Figure 1:**
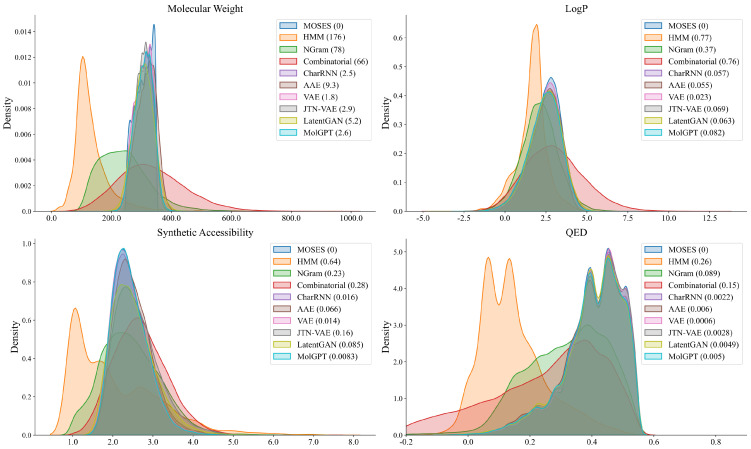
Distribution of chemical properties for the MOSES dataset and sets of generated molecules. The Wasserstein−1 distance to the MOSES test set is denoted in parenthesis. We cover molecular weight, LogP, synthetic accessibility, and QED.

The performance of the various approaches is summarized in Table 1. Our method (base model of cMolGPT) achieved state-of-the-art results in the fraction of valid (Valid), Unique@1k, Unique@10k, fragment similarity, and similarity to the nearest neighbor. The resulting distributions of the four molecular properties in the generated and test datasets are shown in Figure 1. Our model closely matched the real data distribution. This shows that our method is capable of generating drug-like molecules.

### 2.2. Generating Target-Specific Compound Libraries Using Conditional MolGPT

To benchmark the performance of the targeted models, we built a conditional RNN model (denoted as cRNN) by first training a base RNN model on the same MOSES set and fine-tuning it on the target set.

**Figure 2:**
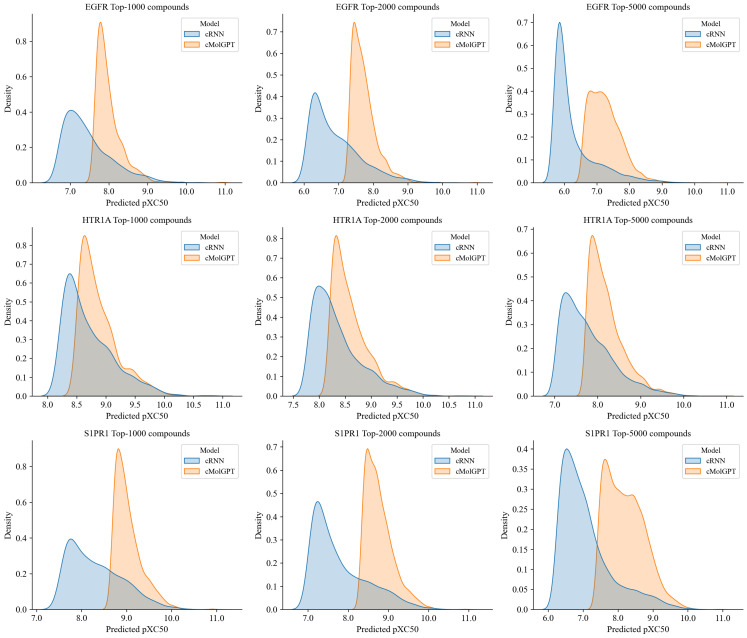
Distributions of predicted activity (pXC50) of the top 1000 (**left**)/2000 (**middle**)/5000 (**right**) compounds from cMolGPT (orange) and cRNN (blue) for the EGFR (**top**), HTR1A (**middle**), and S1PR1 (**bottom**) targets.

We sampled 30,000 compounds from the cMolGPT and cRNN models and the metrics are presented in Table 2. The results demonstrate that the validity of all cases was above 88%, and the uniqueness of 10 k valid compounds was 94%, 90%, and 83% for EGFR, HTR1A, and S1PR1, respectively, which were higher than the values of the cRNN model, except for S1PR1. Moreover, in terms of novelty, the values were 90%, 78%, and 68% for EGFR, HTR1A, and S1PR1 respectively, demonstrating that cMolGPT significantly outperformed the cRNN model. This shows that cMolGPT is not only able to generate valid compounds but also design novel molecules, which is critical for de novo drug design. Furthermore, we used the QSAR models to predict the activity of all the generated valid compounds. We ranked the compounds based on the predicted values and plotted the activity distribution of the top 1000/2000/5000 most active ones, as shown in Figure 2. The results show that the distributions of the predicted activity of the compounds from cMolGPT were significantly better than those from cRNN across all three targets. This highlights that cMolGPT is more capable of generating compounds that are predicted to be active than cRNN.

**Figure 3:**
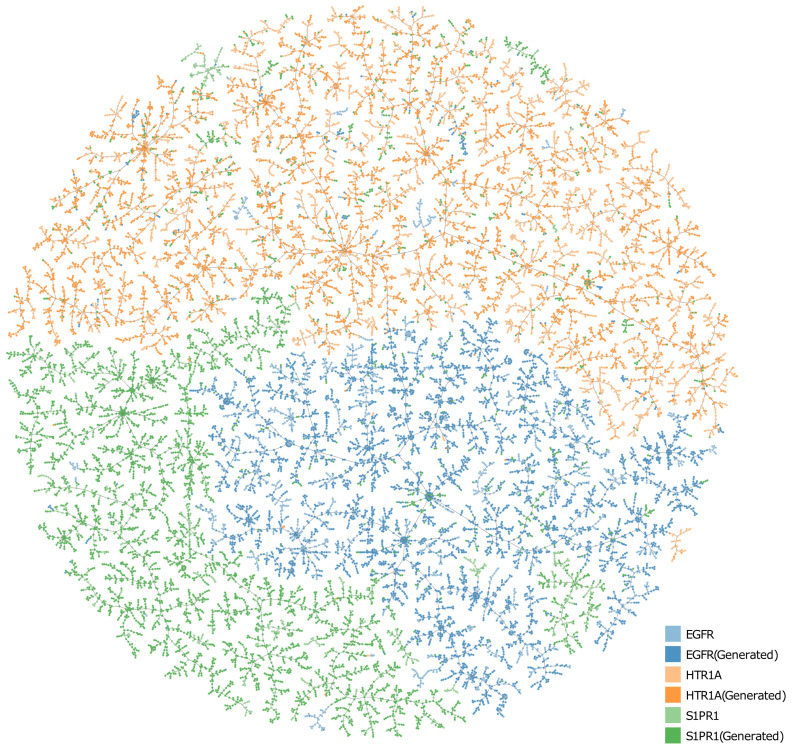
TMAP of the target-specific molecules (dark colors) generated by the proposed cMolGPT model, as well as the ground-truth target-specific molecules (light colors).

Moreover, we evaluated the model’s ability to generate target-specific compounds by visualizing the chemical space. The hypothesis was that compounds potentially interacting with the same protein target would populate the same sub-chemical space. To evaluate overlapping in the chemical space, we selected the top 5000 predicted active compounds for each target, and then the MinHash fingerprint [34] vectors were calculated for the generated compounds, together with the real compounds from the training dataset (the training dataset here refers to the fine-tuned dataset). We used Tree MAP (TMAP) [35] to construct the 2D projections. These projections are illustrated in Figure 3. Each point corresponds to a molecule and is colored according to its target label. The dark and light colors represent the generated compounds and training set compounds, respectively. The visualization of the chemical space shown in Figure 3 demonstrates that the generated target-specific molecules and real target-specific molecules occupied the same sub-chemical space. These results show that our cMolGPT model can generate compounds that are similar to the ones in the training set but are still novel structures.

**Figure 4:**
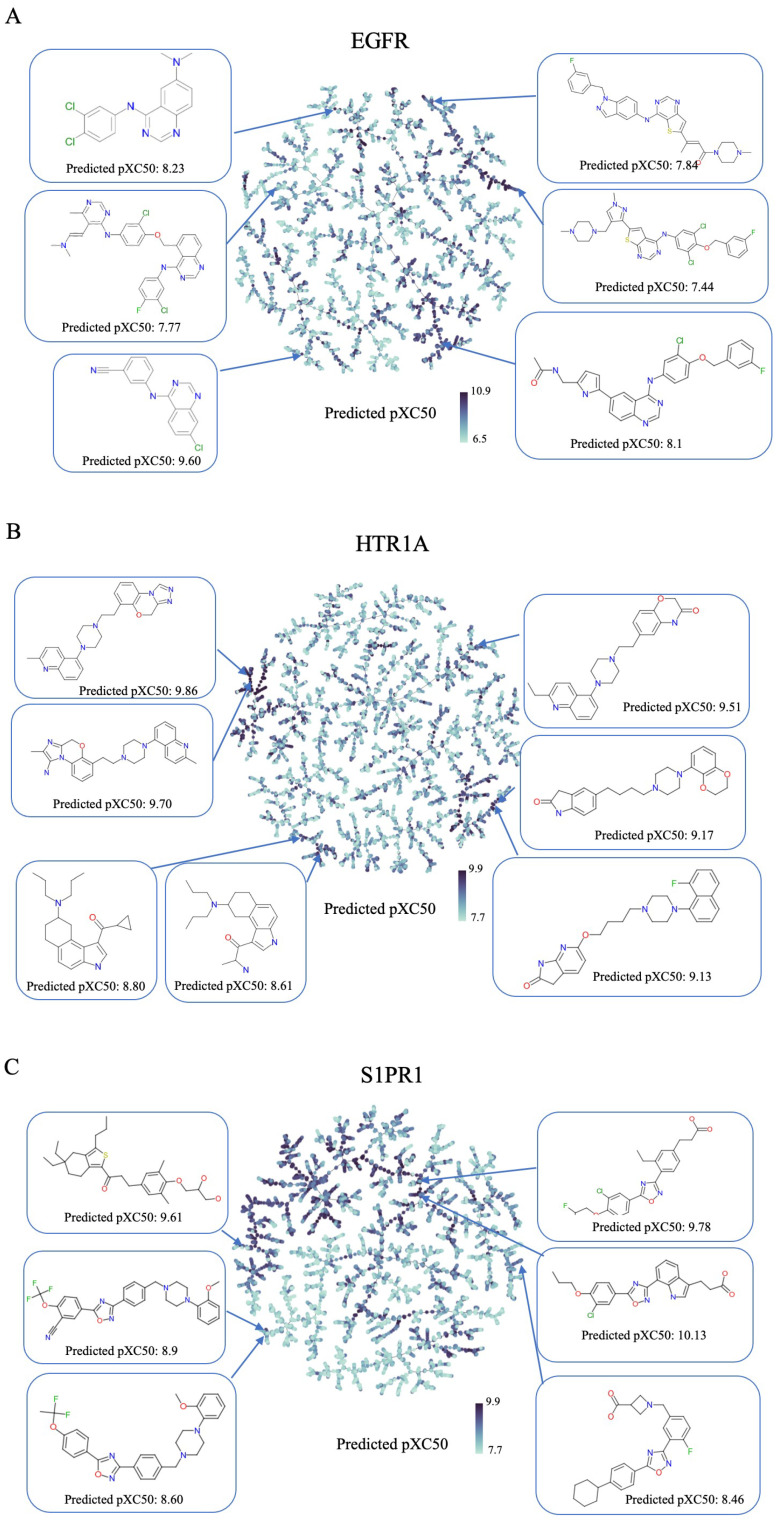
TMAP of the top 5000 generated compounds. (**A**) EGFR, (**B**) HTR1A, (**C**) S1PR1.

Furthermore, we conducted a comprehensive study to evaluate the quality of the generated compounds. A tree-map analysis using MinHash fingerprints was performed to assess the activity of the generated compounds for each target (Figure 4). As seen in this figure, each compound generated by *cMolGPT* is colored according to the predicted pXC50 value, and some structures predicted to have high pXC50 values are indicated. Interestingly, the compounds with high predicted pXC50 values are located in different branches of the tree. This emphasizes that cMolGPT is capable of generating new active series compounds for the target. Additionally, we investigated the properties of the generated compounds for each target. We calculated five physical-chemical properties (molecular weights, TPSA, LogP, HDB, and HBA), QED, and synthetic accessibility (SA) scores for the top 5000 generated compounds and present their maximum and minimum values in Table 3. The results show that our model can generate compounds with a wide range of physical-chemical properties. Moreover, we investigated how many compounds were in the good drug-like property range. We defined a “good” range for each property and present the percentages of generated molecules that fall into that range in Table 4. Most of the sampled compounds have good physical-chemical properties, which demonstrates that *cMolGPT* is capable of generating drug-like compounds.

## 3. Methods and Materials

### 3.1. Problem Formulation

Generative Molecular Design. Given a set of chemical structures of drug D, we aim to learn from D and generate a set of novel chemical structures $\hat{D}$. $\hat{D}$ has to contain valid drug-like structures and must perform well on specific metrics used to quantitatively assess the quality of these novel chemical structures.

Conditional Molecular Design. Appropriate tuning of the binding affinity is a primary objective in molecular design and we aim to support the conditional generation of novel molecules that possess activity against a target protein.

In the scope of this paper, we embed the target protein as a condition. Given a condition (e.g., target) *c*, we aim to generate a set of compounds ${\hat{D}}_{c}$ that are more likely to possess activity against the target protein.

### 3.2. Transformer

RNN-based methods such as seq2seq with attention have achieved excellent performance in sequential modeling (e.g., machine translation) but the recurring nature of RNNs hinders its parallelization, thus making it hard to effectively model long sequences. A Transformer [22] is proposed to address sequential modeling using attention, which is suitable for parallelization and performs well in handling long input sequences [36,37]. In addition to sequence-to-sequence modeling, the Transformer also works well for decoder-only sequence transduction [38].

Many neural sequence transduction models consist of an encoder and a decoder. The encoder first takes a sequence of tokens $\left(x_{1},\ldots ,x_{m}\right)$ and transforms them into a sequence of latent representations $z=(z_{1},\ldots ,z_{m})$ (e.g., memories). The decoder will generate an output sequence $\left(t_{1},\ldots ,t_{n}\right)$ by conditioning on z one element at a time. An intuitive way of sampling sequences is auto-regressive generation [39], which means consuming all the previously generated tokens while generating the next one. Although the traditional Transformer model has an encoder–decoder structure, we define our de novo SMILES generation task as a conditional generator and we use a decoder-only design. Nevertheless, in this paper, we discuss the complete design of the Transformer model.

An attention mechanism mimics the process of querying a set of key–value pairs, where the output is a weighted sum over the values and each weight is based on the matching of the key and query. The multi-head attention projects the keys, values, and queries *h* times and performs attention in parallel. The formal definition of multi-head attention is as follows:

Multi-Head Attention (MHA). We first define some annotations: query matrices $Q_{i}=QW_{i}^{Q}$, key matrices $K_{i}=KW_{i}^{K}$, and value matrices $V_{i}=VW_{i}^{V}$ (i=1,…,h).

$$\begin{array}{cc} O_{i} & =Attention(Q_{i},K_{i},V_{i})\\ \; & =softmax\left(\frac{Q_{i}K_{i}^{T}}{\sqrt{d_{k}}}\right)V_{i} \end{array}$$

$$\begin{array}{c} MultiHeadAttention\left(Q,K,V\right)=CONCAT\left(O_{1},\ldots ,O_{h}\right)W^{O} \end{array}$$

$W_{i}^{Q}\in R^{d_{model}\times d_{k}},W_{i}^{K}\in R^{d_{model}\times d_{k}}$$W_{i}^{V}\in R^{d_{model}\times d_{v}}$ are learnable parameters.

Encoder. The encoder has a stack of identical layers. Each layer has two sub-layers: a multi-head attention component, followed by a feed-forward network. A residual connection is deployed around each of the two sub-layers, followed by layer normalization.

Decoder. The decoder also has a stack of identical layers. Each layer has three sub-layers. Two of them are the same as in the encoder, and the third performs multi-head attention over the output (e.g., latent representations z) of the encoder.

We propose to use a decoder-only Transformer to generate molecules in SMILES format. The token-wise generation is performed in an auto-regressive manner. At each step, the decoder consumes the previously generated tokens as input while generating the next token. The proposed model is pre-trained on a large-scale SMILES dataset to learn a parametric probabilistic distribution over the SMILES vocabulary space and ensure that the generated outputs are in compliance with the SMILES grammar (e.g., atom type, bond type, and size of molecules) (see Section 3.3). Then, the conditional generation is enforced by feeding target-specific embeddings to the multi-head attention component of the Transformer (Section 3.4).

### 3.3. Unsupervised Generative Pre-Training

Many deep learning tasks rely on supervised learning and human-labeled datasets. For instance, the sequence-to-sequence [40] (or seq2seq) model has enjoyed massive success in many natural language processing applications, and the models are usually trained end-to-end with a large number of training pairs (e.g., article–summary pairs for text summarization).

However, in the chemical space, given a large amount of unlabeled data but limited labeled data, we form an unsupervised learning task for drug discovery to overcome the challenge of expensive and hard-to-manage human labeling. Instead of a seq2seq encoder–decoder model, we consider a decoder-only generative model and create a task to predict the next token given previously generated tokens.

GPT [26] and GPT2 [27] have achieved great success in language generation. Specifically, GPT2 learns natural language by predicting the next word given the previous words. Inspired by the success of unsupervised Transformers in NLP (e.g., GPT [26] and GPT2 [27]), we propose to use the Transformer-based model for small-molecule optimization and hit finding. Since a Transformer-based model works well for natural language applications such as writing assistants and dialogue systems, we are optimistic about its capability of generating drug-like SMILES sequences.

**Figure 5:**
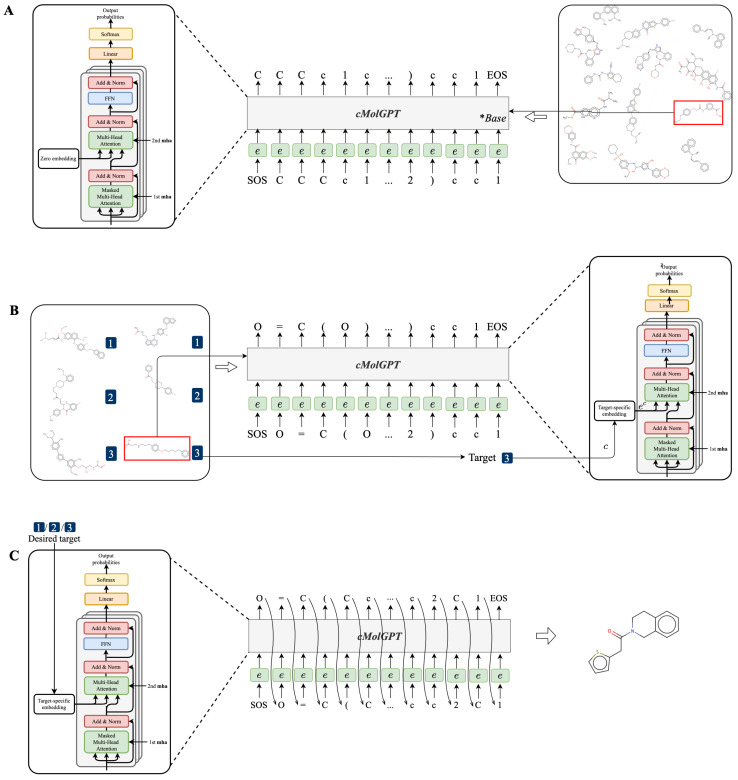
The workflow of our cMolGPT design. (**A**) Pre-training the cMolGPT architecture. (**B**) Fine-tuning the cMolGPT architecture. (**C**) Target-specific conditional molecular generation.

We create our drug discovery task by leveraging the SMILES sequential structure and transforming it into a sequential generation. As shown in Figure 5A, we formalize our task to predict the next token given the previous tokens. The sequential dependencies are trained to mimic the structures observed in the training set and follow the SMILES grammar. During auto-regressive generation, the sampling should be able to produce variations that were not previously observed.

### 3.4. Conditional Generative Pre-Trained Transformer

Our decoder-only design is able to memorize drug-like structures during pre-training. By conditioning on pre-defined conditions, we are able to further confine the search space and sample drug-like structures with the desired properties.

Our decoder learns a parametric probabilistic distribution over the SMILES vocabulary space, conditioned on the target condition denoted as *c*, as well as the previously generated tokens. At the *i*-th step, the decoder produces a probabilistic distribution for the new token, with attention given to the embeddings of the previously generated tokens $\left[e_{1}^{t},\ldots ,e_{i-1}^{t}\right]$ and the target-specific embedding $e^{c}$.

$$\begin{array}{cc} z^{\left(0\right)} & =\left[e_{1}^{t};e_{2}^{t};\ldots ;e_{i-1}^{t}\right]\\ {\bar{z}}^{\left(l-1\right)} & =\mathrm{LN}(z^{\left(l-1\right)}+\mathrm{MHA}\left(z^{\left(l-1\right)},z^{\left(l-1\right)},z^{\left(l-1\right)}\right))\\ {\bar{z}}^{\left(l\right)} & =\mathrm{LN}({\bar{z}}^{\left(l-1\right)}+\mathrm{MHA}\left({\bar{z}}^{\left(l-1\right)},e^{c},e^{c}\right))\\ z^{\left(l\right)} & =\mathrm{LN}({\bar{z}}^{\left(l\right)}+\mathrm{FFN}\left({\bar{z}}^{\left(l\right)}\right)) \end{array}$$

*LN* denotes the layerNorm, *MHA* denotes the multi-head attention, and *FFN* denotes the feed-forward network. Note that we use masks by multiplying masked positions with negative infinity to avoid attending to the masked positions. By attending to the previously generated tokens, we maintain the structural consistency with the SMILES grammar. In other words, we make sure the generated sequence is drug-like based on the memorization acquired during training.

Incorporating target information. One of the major challenges of our task is to generate target-specific SMILE sequences. The model needs to not only memorize a valid drug-like structure but also be able to memorize and generate target-specific information. The challenge lies in capturing target-specific information and further target-specific generation. We propose to leverage the multi-head attention in the Transformer decoder and impose the target-specific embeddings to the keys and values of the attention operations. We refer to our Transformer model with imposed conditional embeddings (e.g., target-specific embeddings) as cMolGPT.

This architecture was inspired by the success of the Transformer encoder–decoder structure in sequence-to-sequence translation (e.g., machine translation), where the encoder memorizes the input sentence and stores it in “memories” and the decoder attends to the previously generated tokens (first MHA) and then performs multi-head attention over the output “memories” of the encoder (second MHA). When attending to the “memories” of the encoder using the second multi-head attention, the queries are from the first multi-head attention and the values and keys from the “memories” of the encoder. The idea is to enable the decoder to attend over the input sequence [22].

We enable the SMILES sequence generation to be conditioned on a specific target by feeding target-specific embeddings (denoted as $e^{c}$) to the decoder-only Transformer (shown in Figure 5B). We use target-specific embeddings as the keys and values within the second MHA, which allows each position of the decoder to attend to the target-specific embeddings, ensuring that the subsequent token generations are conditioned on these embeddings. It is worth noting that our target-specific design is orthogonal to the decoder and can be easily removed by setting the condition embeddings as zero embeddings.

As shown in Figure 5, instead of fetching memory from an encoder, our decoder only initializes the memory based on the condition embeddings. For base model pre-training, $e^{c}$ is initialized with zero embeddings; when targets are involved, $e^{c}$ is initialized with target-specific embeddings.

### 3.5. Workflow for Training and Sampling of the cMolGPT

As illustrated in Figure 5, the training process of our task can be summarized as follows:

1. We first pre-trained the base model of cMolGPT by setting the target-specific embeddings as zero embeddings (without feeding target-specific information) on the MOSES database, as shown in Figure 5A. We did not place any target constraints on the sequential generation and solely focused on learning the drug-like structure from the data.
2. To incorporate the target-specific information, we fine-tuned cMolGPT using <compound, target> pairs, which involved enforcing the conditions of the corresponding target by feeding target-specific embeddings to the attention layer as “memories”, as shown in Figure 5B. We used data from [33], where each SMILES sequence is manually tagged with a target (e.g., target proteins), indicating the specific physicochemical property of the small molecule.
3. We generated a drug-like structure by auto-regressively sampling tokens from the trained decoder, as shown in Figure 5C. Optionally, we enforced the desired target by incorporating a target-specific embedding. The new generation will condition the target-specific information and likely has the desired property. The target-specific embeddings are denoted as $e^{c}$.

### 3.6. Likelihood of Molecular Sequential Generation

An intuitive idea for likelihood estimation of SMILES string sampling is to use a Negative Log-Likelihood (NLL) loss. We propose a conditional design by forcing the *values* and *keys* of multi-head attention to be the generative condition (denoted as c). Overall, our conditional NLL is as follows with the initial state set to *c*:

$$\begin{array}{cc} NLL(S|c)=- & \left[lnP\left(t_{1}|c\right)+\sum_{i=2}^{N}lnP\left(t_{i}|t_{1:i-1},c\right)\right] \end{array}$$

where *c* represents the generative condition (e.g., target protein), *S* is a SMILES sequence with length *N*, and $t_{i}$ is the *i*-th token.

### 3.7. Molecular Dataset and Target-Specific Dataset

We used the MOSES molecular dataset from Polykovskiy [11] to perform unsupervised pre-training of the proposed *cMolGPT*, with the target-specific embedding initialized as zero. The dataset contains 1,760,739 drug molecules extracted from the ZINC clean Lead Collection [41], including 1,584,664 training molecules and 176,075 testing molecules. We adopted the same train/test split used in [11]. Target-specific molecular datasets from [33] were used for training our target-specific *cMolGPT*, which contain 1381, 795, and 3485 active molecules corresponding to the EGFR, S1PR1, and HTR1A target proteins, respectively.

### 3.8. ML-Based QSAR Model for Active Scoring

To evaluate the model’s ability to generate active target-specific compounds, we constructed a regression-based QSAR (quantitative structure–activity relationship) model for each target. The molecular datasets with activity data for each target were acquired from ExCAPE-DB [42] and included 5181, 6332, and 1400 molecules corresponding to the EGFR, HTR1A, and S1PR1 target proteins, respectively. We trained a LightGBM model [43] to predict activity using 2533 molecular features, including a 2048-length FCFP6 fingerprint, 166-length MACCSkeys, and 319 RDKit Molecular Descriptors. We present the Pearson correlation and RMSE on the test dataset in Table 5. The Pearson correlations of the QSAR models on the test data were all higher than 0.75, demonstrating that our QSAR models can benchmark the activity of the generated compounds.

## 4. Conclusions

In this study, we present a Transformer-based random molecular generator and compare it with several baseline models using standard metrics. We demonstrate that the Transformer-based molecular generator can achieve state-of-the-art performance in generating drug-like structures. To incorporate the protein information, we present a target-specific molecular generator by feeding the target-specific embeddings to a Transformer decoder. We apply the method on three target-biased datasets (EGFR, HTR1A, and S1PR1) to evaluate the ability of cMolGPT to generate target-specific compounds and compare it with a conditional RNN. Our results demonstrate that the sampled compounds from the model are predicted to be more active than those from the cRNN across all three targets. Additionally, we visualize the chemical space, and the generated novel target-specific compounds largely populate the original sub-chemical space. In summary, these results demonstrate that our cMolGPT model can be a valuable tool for de novo drug design.

## Figures and Tables

**Table 1:** Performance metrics for baseline models: fraction of valid molecules, fraction of unique molecules from 1000 and 10,000 molecules. Fragment similarity (Frag) and similarity to the nearest neighbor (SNN)—results for the random test set (Test) and scaffold split test set (TestSF). * indicates the base model of cMolGPT.

Model				Frag		SNN	
	Valid	Unique@1k	Unique@10k	Test	TestSF	Test	TestSF
HMM	0.076	0.623	0.567	0.575	0.568	0.388	0.38
NGram	0.238	0.974	0.922	0.985	0.982	0.521	0.5
Combinatorial	1.0	0.998	0.991	0.991	0.99	0.451	0.439
CharRNN	0.975	1.0	0.999	1.0	0.998	0.601	0.565
AAE	0.937	1.0	0.997	0.991	0.99	0.608	0.568
VAE	0.977	1.0	0.998	0.999	0.998	0.626	0.578
JTN-VAE	1.0	1.0	1.0	0.997	0.995	0.548	0.519
LatentGAN	0.897	1.0	0.997	0.999	0.998	0.538	0.514
***cMolGPT*** *	0.988	**1.0**	0.999	**1.0**	**0.998**	0.619	**0.578**

Top performed method in each metric is bold.

**Table 2:** Evaluation metrics: fraction of valid molecules, fraction of unique molecules from 10,000 molecules, and novelty (fraction of molecules not present in the training set).

Target	Model	Valid	Unique@10k	Novel
EGFR	cRNN	0.921	0.861	0.662
	cMolGPT	0.885	**0.940**	**0.898**
HTR1A	cRNN	0.922	0.844	0.498
	cMolGPT	0.905	**0.896**	**0.787**
S1PR1	cRNN	0.926	0.861	0.514
	cMolGPT	0.926	0.838	**0.684**

Top performed method in each metric is bold.

**Table 3:** Physical-chemical properties of generated compounds.

	${\mathrm{MW}}_{\min}$	${\mathrm{MW}}_{\max}$	${\mathrm{TPSA}}_{\min}$	${\mathrm{TPSA}}_{\max}$	${\mathrm{LogP}}_{\min}$	${\mathrm{LogP}}_{\max}$	${\mathrm{HBD}}_{\min}$	${\mathrm{HBD}}_{\max}$	${\mathrm{HBA}}_{\min}$	${\mathrm{HBA}}_{\max}$
EGFR	215.07	781.24	21.06	168.92	−0.82	12.15	0.00	8.00	3.00	14.00
HTR1A	176.09	664.35	3.24	157.04	−0.85	8.92	0.00	6.00	1.00	12.00
S1PR1	263.22	716.17	6.48	227.06	−2.56	13.25	0.00	8.00	1.00	14.00

**Table 4:** Percentages of generated compounds in good drug-likeness range.

	MW ([200, 500])	TPSA ([20, 130])	LogP ([−1, 6])	HBD ([ , 5])	HBA ([ , 10])	QED ([0.4, ])	SA ([ , 5])
EGFR	57.94%	96.68%	62.38%	99.38%	98.08%	39.02%	99.92%
HTR1A	93.80%	96.54%	91.76%	99.96%	99.72%	86.52%	99.84%
S1PR1	72.12%	81.62%	80.26%	97.66%	96.46%	34.06%	99.96%

**Table 5:** Target datasets and the performance of the QSAR models. The active compounds are used for training target-specific generative models. The QSAR models are trained on both active and non-active compounds for each target. R: Pearson correlation; RMSE: root mean square error.

Target				QSAR	
	# of Active Mols	# of Mols	pXC50	R	RMSE
EGFR	1381	5181	6.29±1.39	0.843	0.588
HTR1A	3485	6332	7.33±1.23	0.763	0.631
S1PR1	795	1400	7.25±1.58	0.825	0.779

# refers to number sign.

## Data Availability

Not applicable.

## Sample Availability

The source code and samples of the compounds are available at https://github.com/VV123/cMolGPT (accessed on 23 May 2023).
